# Unconventional seed-mediated growth of ultrathin Au nanowires in aqueous solution†
†Electronic supplementary information (ESI) available. See DOI: 10.1039/c5sc02337h


**DOI:** 10.1039/c5sc02337h

**Published:** 2015-07-20

**Authors:** Bo Li, Beibei Jiang, Haillong Tang, Zhiqun Lin

**Affiliations:** a School of Materials Science and Engineering , Georgia Institute of Technology , Atlanta , GA 30332 , USA . Email: zhiqun.lin@mse.gatech.edu; b High Temperature Resistant Polymers and Composites Key Laboratory of Sichuan Province , School of Microelectronics and Solid-State Electronics , University of Electronic Science and Technology of China , Chengdu 610054 , P. R. China

## Abstract

Ultrathin Au nanowires have garnered increasing attention in recent years because of their potential use in a range of applications due to their unique optical properties, conductivity, chemical activity, and discrete plasticity. Herein, we report an unconventional seed-mediated growth of ultrathin Au nanowires induced by hydrophobic molecules. Quite intriguingly, by adding a trace amount of hydrophobic molecules (*i.e.*, toluene or chloroform) to the Au growth solution conventionally used for the growth of Au nanorods with cylindrical CTAB micelles as templates, CTAB-capped ultrathin Au nanowires (*i.e.*, water-soluble ultrathin Au nanowires) were crafted. Similarly to the growth of Au nanorods, silver ions and Au seeds were crucially required to yield the water-soluble ultrathin Au nanowires. The growth mechanism of these ultrathin nanowires was also explored.

## Abstract 2

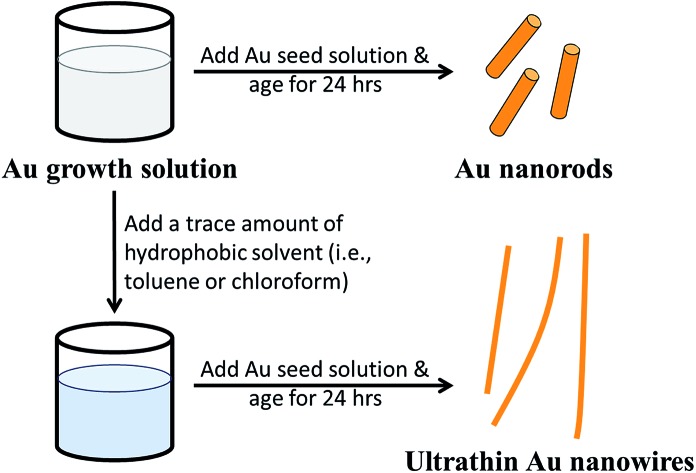
By adding a trace amount of hydrophobic molecules to conventional Au growth solution, CTAB-capped ultrathin Au nanowires were crafted.

## Introduction

Controlling the size, shape, composition, and structure of metal nanocrystals is of fundamental and technological importance as the optical, electrical, and catalytic properties of metal nanocrystals depend heavily on these parameters. In this context, due to the one-dimensional (1D) confinement of electron transport and the high surface area, metallic nanowires possess many intriguing functionalities for use in optical waveguides,^1^,^2^ lasers,^3^ sensors,^4^–^7^ and nanoelectronics.^8^–^11^ Interestingly, among various 1D Au nanostructures, ultrathin Au nanowires offer a wide range of potential applications^12^–^16^ because of their unique optical properties,^17^–^19^ conductivity,^20^–^22^ chemical activity,^23^,^24^ and discrete plasticity.^25^ Notably, much effort has been concentrated on the creation of 1D Au nanorods based on the commonly used cetyltrimethylammonium bromide (CTAB) template strategy.^26^–^28^ The preferential physisorption of CTAB to the {100} and {110} facets facilitates the 1D Au growth along the [110] direction from the prepared Au seeds (*i.e.*, Au nanoparticles).^29^–^31^ Yet such longitudinal growth usually discontinues when the aspect ratio of the length to the diameter of the nanorods reaches 10.^26^,^32^ Even though multistep growth allows for the formation of Au nanorods with an aspect ratio of 20 or higher,^33^–^36^ the diameter of Au nanorods grown from Au seeds is usually larger than 10 nm or even beyond 50 nm,^37^ despite the fact that the diameter of the Au seeds used for the nanorod growth is of much smaller size (*i.e.*, usually <3 nm).^26^ However, it is highly desirable to yield ultrathin Au nanowires with diameters of 2–3 nm for the promising applications noted above.^38^ To the best of our knowledge, there has been no report to date on the synthesis of ultrathin Au nanowires employing the CTAB-templating strategy.

On the other hand, ultrathin Au nanowires have been synthesized *via* an aurophilic reaction by employing oleylamine (OA) as the solvent and reducing agent.^39^ These OA-capped ultrathin Au nanowires, however, are non-water soluble,^40^–^43^ and this limits their use in biomedical applications.^44^ The strong absorption of OA on a Au nanowire surface makes it difficult for further ligand-exchange to yield water-soluble Au nanowires.^45^ Herein, we report an unconventional seed-mediated growth of ultrathin Au nanowires induced by hydrophobic molecules. Quite surprisingly, rather than the commonly obtained Au nanorods, by introducing a small amount of hydrophobic molecules (*i.e.*, toluene or chloroform) into the Au growth solution based on the conventional preparative approach for Au nanorods with cylindrical CTAB micelles as templates, CTAB-capped ultrathin Au nanowires (*i.e.*, water-soluble ultrathin Au nanowires) were yielded. It is interesting to note that there is a certain range of CTAB concentration beyond which no ultrathin Au nanowires can be produced. Moreover, silver ions and Au seeds required for the Au nanorod growth were found to be crucial also in the formation of the CTAB-capped ultrathin Au nanowires. The growth mechanism of such intriguing water-soluble ultrathin Au nanowires, which differed from those formed when using oleylamine (*i.e.*, the non-water-soluble Au nanowires),^39^ was explored.

## Results and discussion

**Fig. 1:**
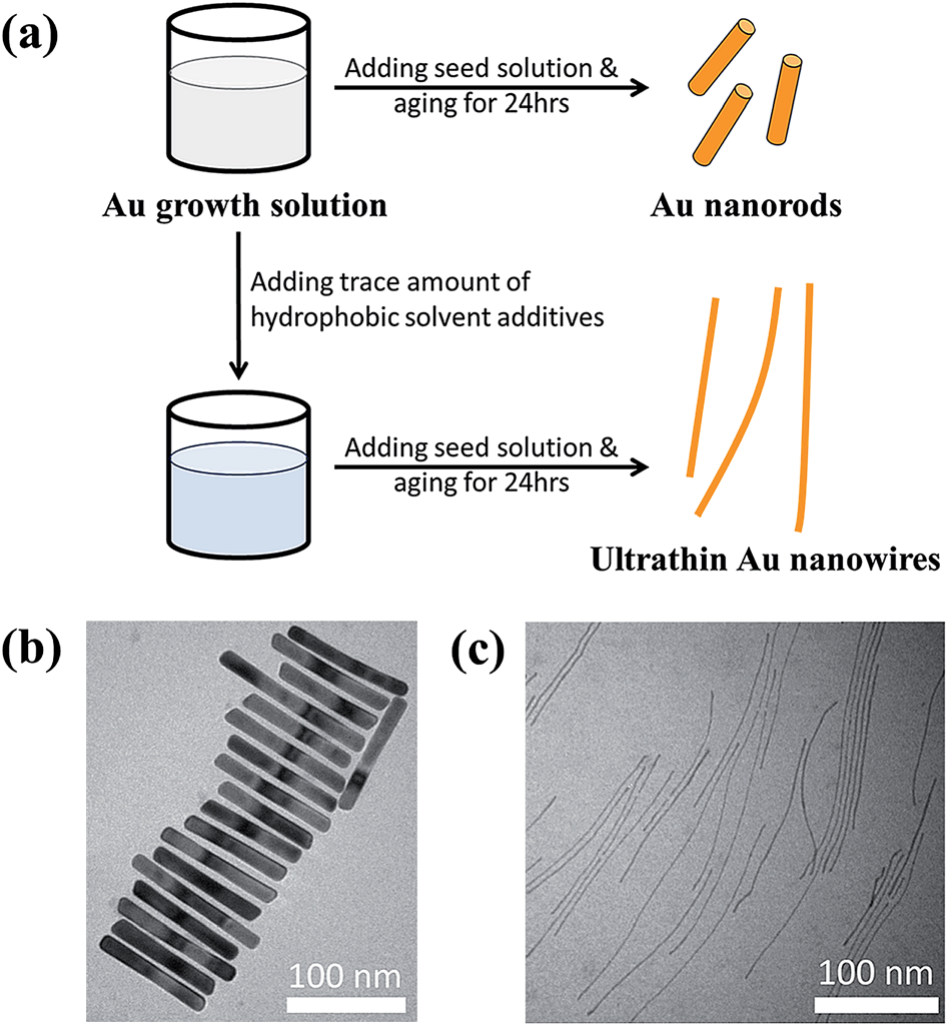
(a) A schematic illustration of the synthesis of Au nanorods in the pure Au growth solution (upper right panel), and of the ultrathin Au nanowires with the introduction of a trace amount of hydrophobic solvent (*i.e.*, toluene or chloroform) into the Au growth solution (lower right panel). (b) The Au nanorods synthesized without the addition of toluene, corresponding to the upper right panel in (a). (c) Ultrathin Au nanowires formed with the addition of toluene, corresponding to the lower right panel in (a).

Fig. 1a compares the effect of introducing a trace amount of hydrophobic solvent (*i.e.*, toluene) into the Au growth solution. The Au seeds were prepared according to the literature.^31^ Unless noted otherwise, the Au growth solution was prepared based on Zubarev's method as follows:^32^ 10 ml of 0.1 M CTAB aqueous solution was mixed with 0.5 ml of 10 mM HAuCl_4_ solution, and 40 μl of 0.1 M AgNO_3_ solution. Subsequently, 0.5 ml of 0.1 M hydroquinone (as the reducing agent) aqueous solution was added and stirred until the mixed solution became transparent. Without the addition of toluene to the Au growth solution, Au nanorods were produced in high yield after the addition of the Au seed solution (upper panels in Fig. 1a and b, and Fig. S1†). However, upon the addition of a trace amount of toluene (100 μl) to the Au growth solution and being mixed well, followed by adding the Au seed solution (see ESI†), the final solution remained colorless after a one-day reaction. Surprisingly, from this transparent solution, ultrathin Au nanowires were observed as revealed from the TEM measurement (Fig. 1c). It is noteworthy that in stark contrast to non-water soluble ultrathin Au nanowires synthesized using oleylamine as in several previous studies,^46^ which are often self-assembled side-by-side, in this work the resulting CTAB-capped ultrathin Au nanowires laid freely on the TEM grid because of the repulsion between the adjacent nanowires due to the presence of the positively charged CTAB on the surface of the nanowires.

**Fig. 2:**
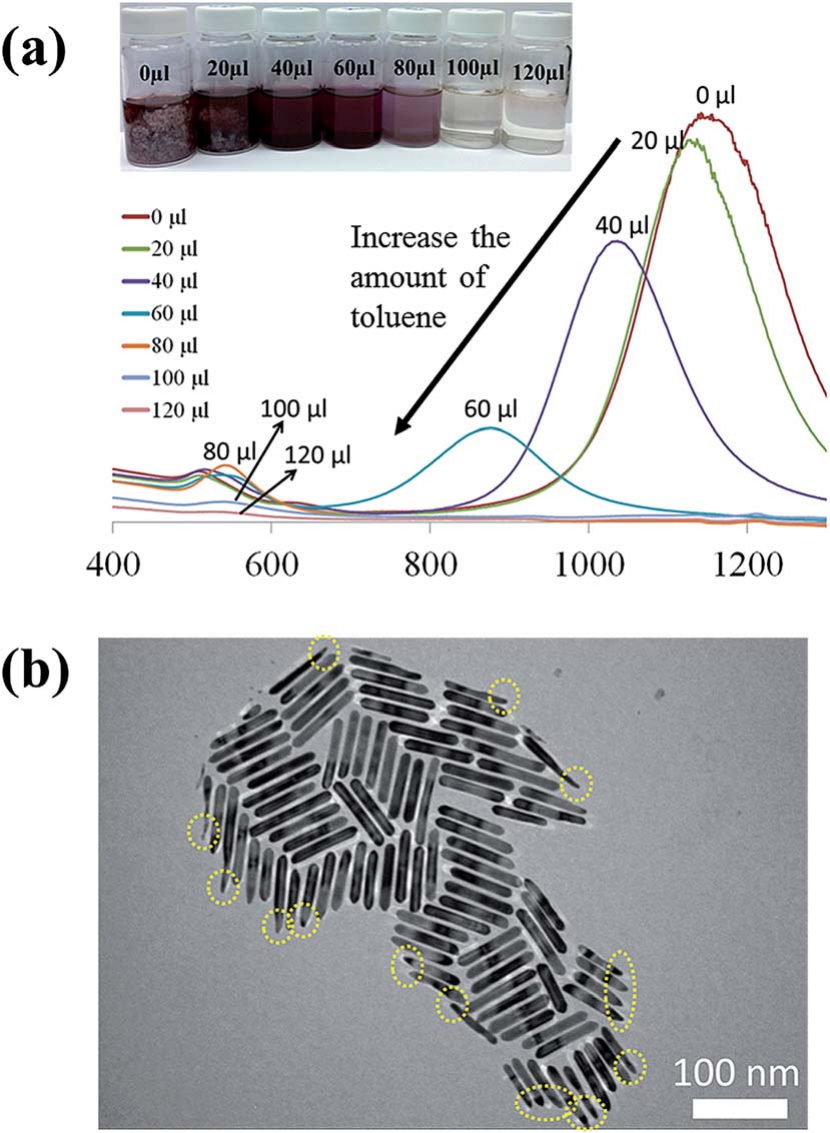
(a) UV-vis spectra and the corresponding digital images (insert) of the solutions with the introduction of varied amounts of toluene (*i.e.*, 0 μl, 20 μl, 40 μl, 60 μl, 80 μl, 100 μl and 120 μl). The Au growth solution was prepared by mixing 10 ml of 0.1 M CTAB aqueous solution with 0.5 ml of 10 mM HAuCl_4_ solution, 40 μl of 0.1 M AgNO_3_ solution and 0.5 ml of 0.1 M hydroquinone aqueous solution. Subsequently, toluene was introduced and well mixed with the growth solution. Finally, 80 μl of the Au seed solution was added to the abovementioned mixed solution and allowed to react for 24 h to yield the final solution. (b) Au nanorods obtained from the solution with the introduction of 40 μl toluene. Some of the Au nanorods with tapered ends are marked with dotted circles.

To further scrutinize the influence of toluene on the formation of ultrathin Au nanowires, we first prepared the growth solution and mixed it with a varied amount of toluene (*i.e.*, 0 μl, 20 μl, 40 μl, 60 μl, 80 μl, 100 μl, and 120 μl). After that, 80 μl of an Au seed solution was introduced to the abovementioned mixed solution and stirred for 2 min. The solution was then allowed to age for 24 h. The digital images of the solutions containing the final products are shown as insets in Fig. 2a. As the amount of toluene added to the growth solution increased, the brownish-red color (*i.e.*, with 0–40 μl of toluene added) gradually turned into light pink (*i.e.*, with 60–80 μl of toluene added), and eventually became a transparent solution with further addition of toluene (*i.e.*, 100 μl and 120 μl). The color of the solutions upon the addition of toluene from 0 μl (*i.e.*, formation of the Au nanorods) to 40 μl did not have a noticeable change. The UV-vis measurements showed the emergence of two characteristic surface plasmonic resonance peaks, that is, a transverse surface plasmon peak at ∼520 nm and a longitudinal surface plasmon peak at ∼1100 nm, with the addition of 0 μl of toluene, signifying the formation of Au nanorods.^32^ Clearly, the longitudinal absorption peak of the Au nanorods blue-shifted slightly with the increase of toluene from 0 μl to 40 μl (Fig. 2a), which is in good agreement with the color change of the solution (inset in Fig. 2a). Nonetheless, this observation suggested the presence of Au nanorods when the amount of added toluene was at 20 μl and 40 μl. Intriguingly, some Au nanorods created by adding 40 μl of toluene showed tapered ends (marked with the dotted circles in Fig. 2b), indicating that the addition of toluene may lead to the shrinking of the cylindrical CTAB micellar templates at the ends of the nanorods. In addition, the intensity of the longitudinal absorption peak decreased dramatically as the amount of toluene increased from 60 μl to 80 μl and the brownish-red color of the solution gradually disappeared. Further scrutiny from TEM measurements showed that the solution containing 80 μl toluene comprised only a few Au nanorods together with a small amount of short ultrathin Au nanowires (Fig. S2†), indicating that the growth of the Au nanorods was strongly suppressed upon the addition of toluene. Notably, further increase in the amount of toluene (*e.g.*, 100 μl) led to the disappearance of the longitudinal absorption peak over the entire wavelength range (*i.e.*, 400–1200 nm), suggesting that almost no Au nanorods were formed in the solution. Instead, ultrathin Au nanowires were obtained (Fig. S3†). Recently, it was reported that the longitudinal absorption peak of oleylamine-capped ultrathin Au nanowires can exceed more than 1200 nm and even reach up to 10 000 nm, which is beyond the measurable wavelength range using a UV-vis spectrometer,^18^ as a result of their ultrahigh aspect ratios of length to diameter. Thus, in our study the longitudinal absorption peak for the ultrathin Au nanowires cannot be detected with a UV-vis spectrometer (Fig. 2a).

**Fig. 3:**
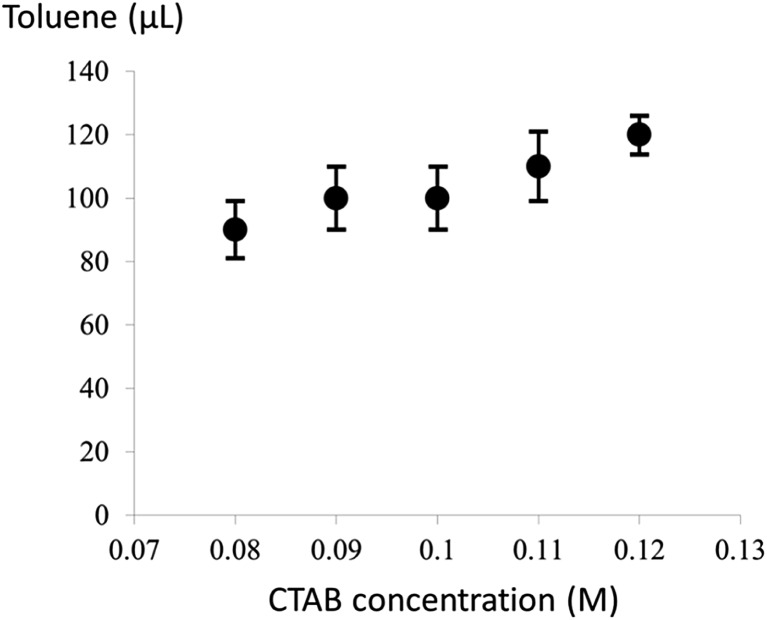
The critical amount of toluene required for the formation of the ultrathin Au nanowires at different CTAB concentrations.

It is noteworthy that the introduction of a trace amount of toluene into the Au growth solution containing CTAB is the key to the formation of the ultrathin Au nanowires in our study. To further explore the synergistic effect of the cylindrical CTAB micellar template and the trace amount of toluene on the creation of the ultrathin Au nanowires, samples with the CTAB concentration ranging from 0.08 M to 0.12 M (*i.e.*, 0.08 M, 0.09 M, 0.10 M, 0.11 M, and 0.12 M) were prepared. Interestingly, the critical amount of toluene required for obtaining the colorless solution slightly decreased from 120 μl to 90 μl as the concentration of CTAB decreased from 0.12 M to 0.08 M (Fig. 3), respectively.

**Fig. 4:**
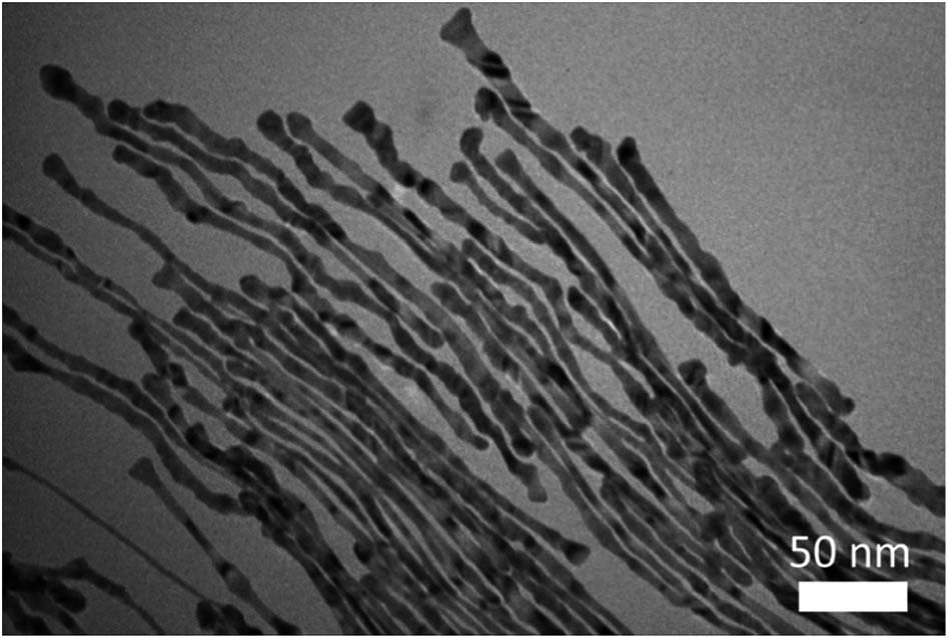
A TEM image of the Au nanowires of larger diameter formed at a CTAB concentration of 0.07 M with the addition of 100 μl of toluene to the Au growth solution prior to the addition of the Au seed solution.

In addition, for CTAB concentrations below 0.08 M, no ultrathin Au nanowires were yielded. For example, Au nanowires with much larger diameters were formed at the CTAB concentration of 0.07 M (Fig. 4 and S4†), and no Au nanowires were observed with a further decrease in the CTAB concentration. Moreover, for low CTAB concentrations (*i.e.*, 0.05 M), Au nanorods can still be obtained without the addition of toluene (Fig. S5†). When the amount of toluene introduced was more than 50 μl, the solution became transparent with no Au nanowires formed in the solution. However, the solution exhibited a dark-red color again with the addition of more than 100 μl of toluene. The corresponding TEM image clearly shows the formation of Au nanorods; however, both the length and diameter of the Au nanorods have a rather wide distribution again (Fig. S6†). The formation of such non-uniform Au nanorods merits a detailed study and will be the subject of future study.

In addition to the CTAB concentration, the effects of the variation in the Au seeds and silver ions (Ag^+^) were also investigated while keeping other experimental parameters constant. Without adding the Au seeds, no ultrathin Au nanowires were found in the prepared solution; even though it was allowed to react for two weeks. Obviously, the Au seeds served as the nucleation sites for the growth of the ultrathin Au nanowires in this work. Moreover, Ag^+^ has been proven to be critical for the Au nanorod growth^26^,^47^ by preventing the Au growth at the {110} and {100} facets and thus promoting longitudinal growth.^48^ Similarly, we found that Ag^+^ was also important for the growth of the ultrathin Au nanowires. Without the presence of Ag^+^, only Au nanoparticles were formed. Furthermore, when the Au growth solution was prepared with Ag^+^ at a relatively low concentration (*i.e.*, 0.04 mM by adding 40 μl of 0.01 M Ag^+^ solution to 10 ml of the Au growth solution), Au nanostructures with irregular shapes were yielded when 100 μl of toluene was added (Fig. S7 and S8†). Finally, without the addition of toluene, short Au nanorods were still observed at such low Ag^+^ concentration (0.04 mM; Fig. S9†).

**Fig. 5:**
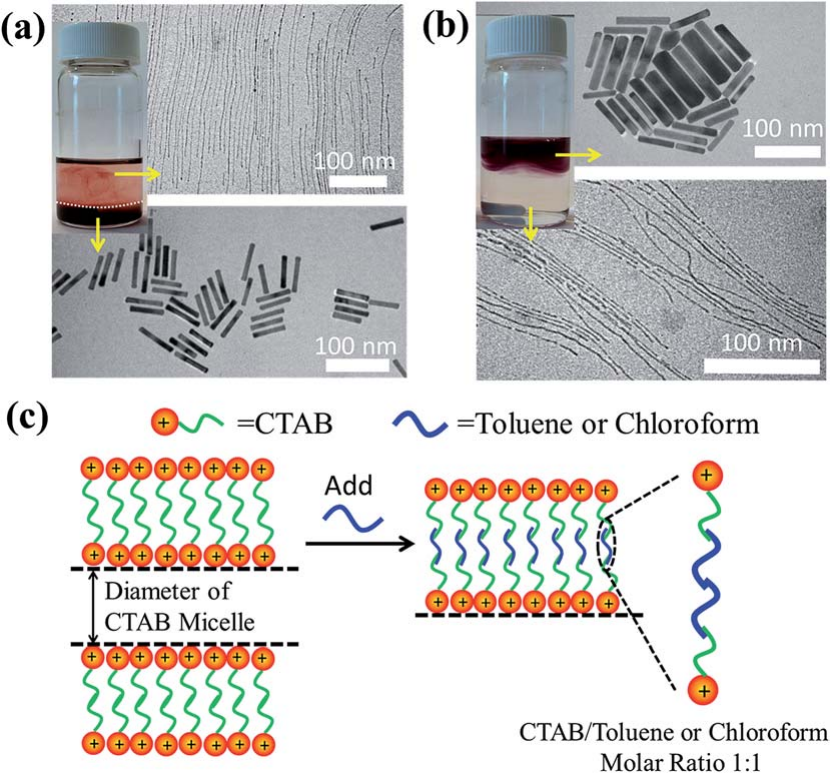
The prepared solutions with layered structures by mixing either (a) toluene or (b) chloroform with the Au growth solution after adding the Au seed solution and gently shaking the mixed solution. (c) The proposed mechanism for the formation of the ultrathin Au nanowires. Hydrophobic molecules (toluene or chloroform) preferably incorporate with the inner hydrophobic chains of the CTAB double layer, thereby leading to a decrease in the inner diameter of the self-assembled cylindrical CTAB micelles, which in turn template the growth of the ultrathin Au nanowires.

We note that the exact role that the hydrophobic molecules played in the growth of the ultrathin Au nanowires remains unclear at this stage. Thus, we first explored where the hydrophobic molecules (*e.g.*, toluene) locate during the growth of the ultrathin Au nanowires, which is crucial for understanding the underlying growth mechanism. To this end, we designed experiments as follows. In contrast to the procedure noted above where toluene was mixed well with the Au growth solution prior to the addition of the Au seed solution, remarkably, when toluene was added to the Au growth solution after adding the Au seed solution, and the mixed solution was simply gently shaken by hand, the solution formed a layered structure (Fig. 5a) (*i.e.*, a dark-red layer on the bottom and a relatively transparent layer on the top). It is worth noting that without the introduction of toluene after adding the Au seed solution, the preparative procedure only led to the formation of Au nanorods. However, with the addition of 100 μl of toluene to the Au growth solution, the as-prepared solution displayed two layers after the 24 h reaction. This is due to the fact that, with gentle shaking, only a part of the toluene was incorporated with CTAB while the rest of the toluene segregated on top of the aqueous solution. Interestingly, Au nanorods were formed on the lower layer (Fig. S10†), which correlated well with the dark-red color observed in the digital image of the solution (inset in Fig. 5a), while the upper layer was primarily composed of the ultrathin Au nanowires (Fig. S11†) as clearly evidenced by the TEM measurements.

On the basis of the observed layered solution, the incorporation between the hydrophobic molecules and CTAB micelles during the growth of the ultrathin Au nanowires may be rationalized as follows (Fig. 5). First, CTAB molecules, which are cationic surfactants, self-assemble into cylindrical micelles in aqueous solution as widely discussed in the literature.^48^ Such cylindrical micelles comprise the CTAB double layers by exposing the cationic ends with the hydrophobic chains inside.^49^ When toluene molecules are mixed with the cylindrical CTAB micelle solution, toluene as a small hydrophobic molecule is preferentially incorporated with the inner hydrophobic chains of the CTAB double layer due to the favorable hydrophobic–hydrophobic interactions.^50^ Such an accommodation of toluene molecules within the CTAB double layer increases the total thickness of the double layer, transforming it into a sandwich-like structure (*i.e.*, toluene molecules situated within the hydrophobic chains of CTAB as depicted in the right panel of Fig. 5c) on each side of the double layer. It is important to note that there were 1 × 10^–3^ moles of CTAB molecules in the as-prepared solution. At the molar ratio of CTAB/toluene = 1 : 1 for the incorporation of toluene inside the CTAB double layer, the corresponding volume of toluene can be estimated to be 106.3 μl (*i.e.*, 
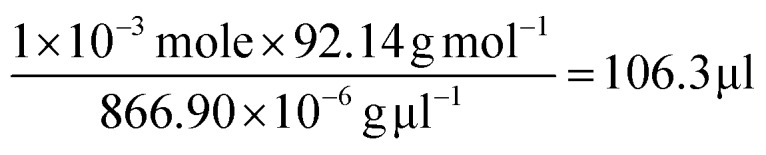
). This correlates well with the critical amount of toluene required for the formation of an ultrathin Au nanowire as described above in Fig. 2 (*i.e.*, 100 μl). The density of toluene is 0.8669 g ml^–1^, lower than that of water. Taken together, the lighter sandwich-like CTAB/toluene/CTAB ligands lifted ultrathin Au nanowires up to the upper layer of the two-layer solution (inset in Fig. 5a). Moreover, it is also noteworthy that the thickness of the top transparent layer (*i.e.*, the toluene layer) was much larger than the bottom layer (*i.e.*, the dark-red layer for the Au nanorod growth), signifying that the majority of the CTAB micelles entered into the upper toluene phase to accommodate the toluene molecules inside even with gentle shaking.

To further verify the CTAB/hydrophobic molecules/CTAB structures discussed above, in addition to toluene, chloroform was also employed as a hydrophobic molecule and added to the Au growth solution. Similar to the case of adding toluene, a transparent solution was also observed when the amount of added chloroform was above a certain value (*i.e.*, 80 μl). The TEM measurement showed the formation of ultrathin Au nanowires (Fig. S12†). Likewise, as the density and molar moss of chloroform are 1.49 g ml^–1^ and 119.38 g mol^–1^, respectively, the corresponding volume of chloroform for incorporating chloroform within 1 × 10^–3^ moles of CTAB was found to be 80.1 μl (*i.e.*, 
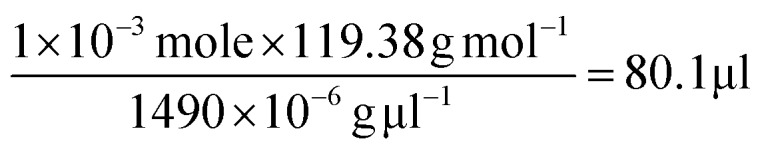
), which also agreed well with the critical volume of chloroform required for the formation of an ultrathin Au nanowire (*i.e.*, 80 μl). Similarly, a layered solution was also seen by mixing chloroform with the Au growth solution after adding the Au seed solution. Intriguingly, the bottom layer of the solution was transparent while the upper layer was dark-red (inset in Fig. 5b), which is completely opposite to the layered solution formed by adding toluene (inset in Fig. 5a). The Au nanorods obtained from the upper layer were not uniform (Fig. 5b). In comparison to the case of toluene (*i.e.*, a dark-red layer on the bottom and a transparent layer on the top), the high density of chloroform (1.48 g ml^–1^; larger than that of water) was responsible for the formation of the switched layered solution (*i.e.*, the colorless layer on the bottom and the dark-red layer on the top). Consequently, the sandwich-like CTAB/chloroform/CTAB-capped ultrathin Au nanowires were produced and sank down to the lower layer of the two-layer solution (inset in Fig. 5b).

Although a detailed understanding for such seed-mediated growth of ultrathin nanowires is yet to be explored, some insights can be gained from the sandwich-like CTAB/hydrophobic molecules/CTAB structure. We suggest two possible reasons for the formation of the ultrathin Au nanowires induced by hydrophobic molecules. First, the incorporation of the hydrophobic molecules with the inner hydrophobic chains of CTAB may lead to a decrease in the inner diameter of the self-assembled cylindrical CTAB micelles, rendering the formation of the ultrathin Au nanowires templated by the shrunk cylindrical micelles. It is noteworthy that hydrophobic molecules may also cause elongation of the self-assembled CTAB micelle.^51^ However, it remains a question whether the shrunk soft cylindrical CTAB micelles were tight enough to constrain the radial growth of the ultrathin Au nanowires. Thus, another possible reason may be that the incorporation between the hydrophobic molecules with CTAB chains facilitated their stronger physisorption to the Au surface,^26^ thereby greatly retarding the radial growth of the Au nanowires. In addition, we note that the oriented-attachment mechanism^17^ (*i.e.*, the ultrathin Au nanowires were formed *via* the oriented attachment and the fusion of the Au seeds) may not be the case as the intermediate products (*e.g.*, irregular chain-like short Au nanowires) were not observed during the ultrathin Au nanowire growth. Clearly, the growth mechanism may be rather complex and thus merits a further study. This is currently under investigation.

## Conclusion

In summary, we have developed a surprisingly simple yet effective strategy for crafting water-soluble ultrathin Au nanowires by introducing a trace amount of hydrophobic molecules (*i.e.*, toluene or chloroform) to the Au growth solution based on a conventional preparative approach for Au nanorods using cylindrical CTAB micelles as templates. The influence of introducing these hydrophobic molecules on the self-assembled CTAB micellar templates, which led to the formation of the ultrathin Au nanowires, was also explored. In principle, such a simple and robust strategy can be extended for the synthesis of a large variety of nanowires that capitalize on CTAB as the surface capping ligands. Furthermore, the ability to create ultrathin Au nanowires may open up exciting opportunities for fundamental studies on their unique physical properties as well as the promising applications in nanoscale photonic, electronic, and optoelectronic devices, sensors, bioimaging, diagnosis, and therapy.

## Supplementary Material

Supplementary information
