# Supplementary material for: Unconventional seed-mediated growth of ultrathin Au nanowires in aqueous solution
Source: Chem Sci. 2015 Jul 20;6(11):6349–54. doi: 10.1039/c5sc02337h (PMC6054065; doi:10.1039/c5sc02337h)
Supplement: Supplementary file 1 [file SC-006-C5SC02337H-s001.pdf]

Electronic Supplementary Material (ESI) for Chemical Science.
This journal is © The Royal Society of Chemistry 2015

Supporting Information

Unconventional growth of seed-mediated ultrathin gold nanowire in aqueous solution

Bo Li,[a] *Beibei Jiang,*[a] *Hailong Tang,*[b] and *Zhiqun Lin*[a]*

a. School of Materials Science and Engineering, Georgia Institute of Technology, Atlanta, GA 30332, USA

b. High Temperature Resistant Polymers and Composites Key Laboratory of Sichuan Province, School of Microelectronics and Solid-State Electronics, University of Electronic Science and Technology of China, Chengdu 610054, P. R. China

*To whom correspondence should be addressed. Email: zhiqun.lin@mse.gatech.edu

Experimental Section

Preparation of Au seed solution. CTAB solution (10 mL, 0.10 M) was mixed with 0.25 mL of 10 mM $HAuCl_4$. 0.6mL freshly prepared 0.01M $NaBH_4$ aqueous solution was then added, resulting in a brownish yellow solution. Notably, the Au seed solution needs to be prepared freshly each time.

Preparation of Au growth solution. CTAB solution (10 mL, 0.10 M) was mixed with 0.5 mL of 10 mM $HAuCl_4$. 40 µl $AgNO_3$ solution (0.1 M) was then added and well mixed. Hydroquinone aqueous solution (0.5mL, 0.1M) was then added to the solution noted above, and stirred until the

solution became colorless and clear. Notably, the time for the solution turning to be colorless depends on the concentration of CTAB. For 0.1 M CTAB, it took around 1 min; and for 0.08M CTAB, it took only several seconds. However, for the solution with lower CTAB concentration (e.g., 0.01 M), the solution remained turbid after the addition of hydroquinone solution.

Synthesis of ultrathin Au nanowires.

(a) Homogeneous solution: A varied amount of toluene (e.g., 0 µl, 10 µl, 20 µl, 40 µl, 60 µl, 80 µl, 90 µl, 100 µl, 110 µl, 120 µl, 130 µl, 140 µl, 150 µl, 200 µl, 400 µl, and 1000 µl) was added to the Au growth solution and stirred for 10 mins. It is worth noting that for the CTAB solution at concentration of 0.1 M, when the amount of toluene was added at or below 100 µl, the solution became clear after well-mixed. However, the addition of more toluene (e.g., 200 µl, 400 µl, and 1000 µl) led to the turbid solutions even after more than 20 mins. After mixing with toluene, a 80-µl Au seed solution was added. The final solution was allowed to age for 24hrs.

(b) Layered solution: A 80-µl Au seed solution was added to the Au growth solution. A varied amount of toluene (e.g., 0 µl, 10 µl, 20 µl, 40 µl, 60 µl, 80 µl, 90 µl, 100 µl, 110 µl, 120 µl, 130 µl, 140 µl, 150 µl, 200 µl, 400 µl, and 1000 µl) was added to the Au growth solution, and gently shaken by hand. The final solution was allowed to age for 24hrs. It should be noted that the Au seed solution should be freshly prepared every time as the Au seed solution from a long-time storage may result in large percentage of Au nanoparticles formed in solution.

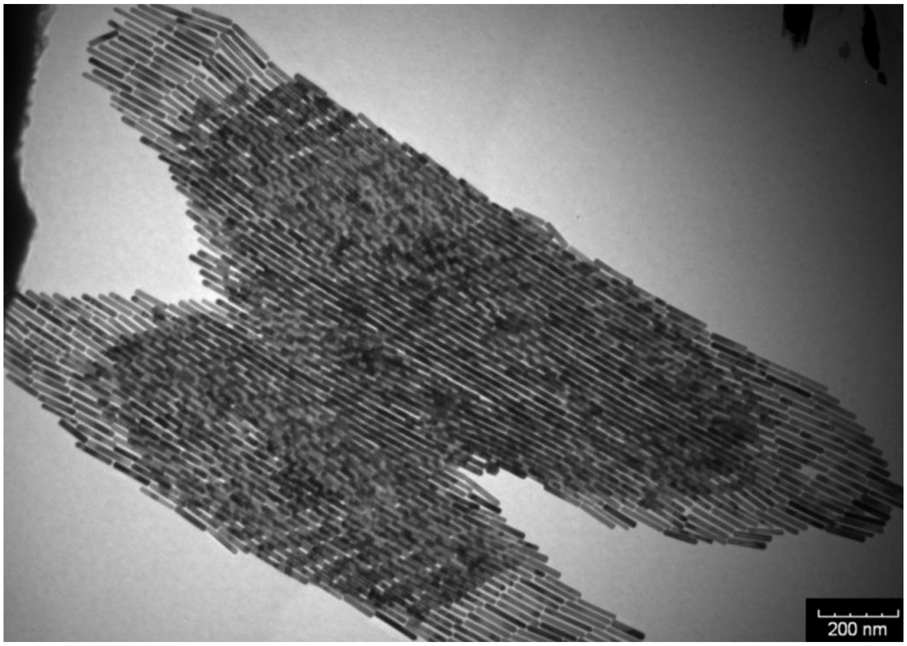

Figure S1. TEM image of Au nanorods with nearly 100% yield synthesized without the addition of hydrophobic solvent (i.e., toluene).

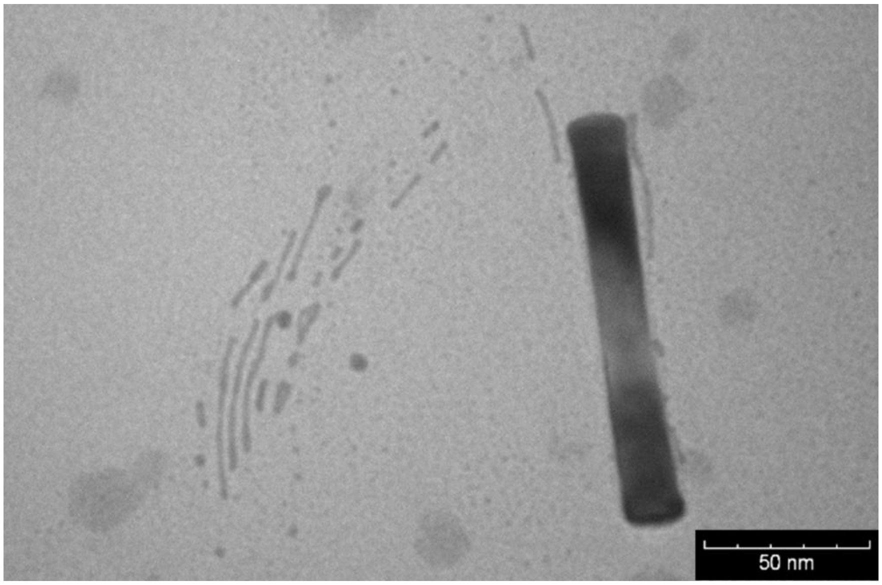

Figure S2. TEM image of one Au nanorod together with a small amount of short ultrathin Au nanowires. An 80-µl toluene was added to the Au growth solution and well mixed prior to the addition of the Au seed solution.

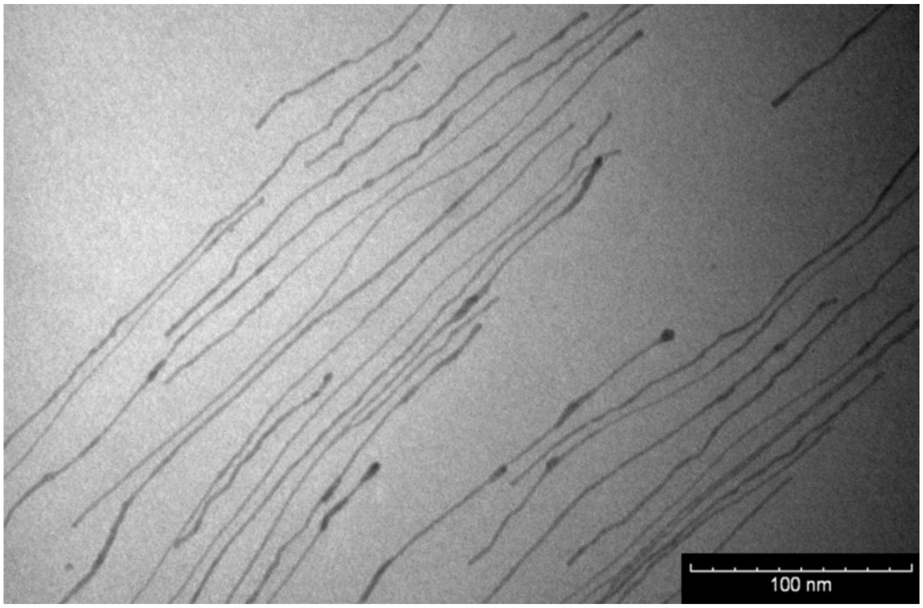

Figure S3. TEM image of ultrathin Au nanowire obtained from the solution with the addition of 100-µl toluene to the Au growth solution and well mixed prior to the addition of the Au seed solution.

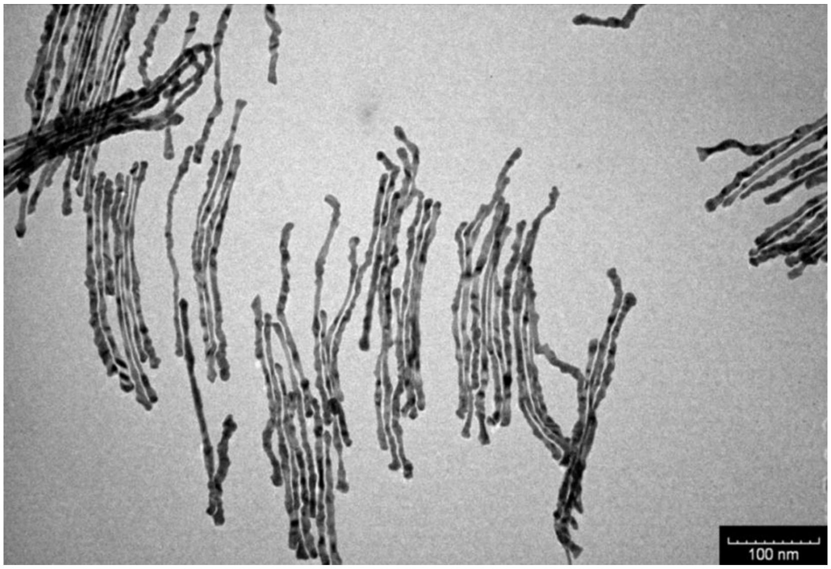

Figure S4. TEM image of Au nanowires of larger diameter formed at CTAB concentration of 0.07M with the addition of 100-µl toluene to the Au growth solution prior to the addition of the Au seed solution.

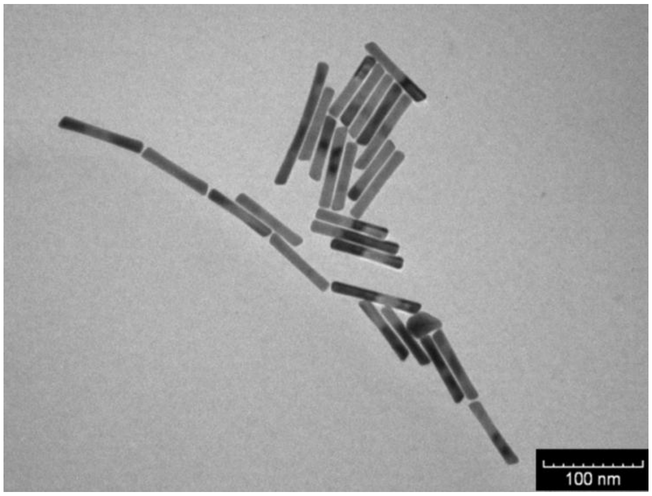

Figure S5. TEM image of Au nanorods formed without the addition of toluene at CTAB concentration of 0.05M.

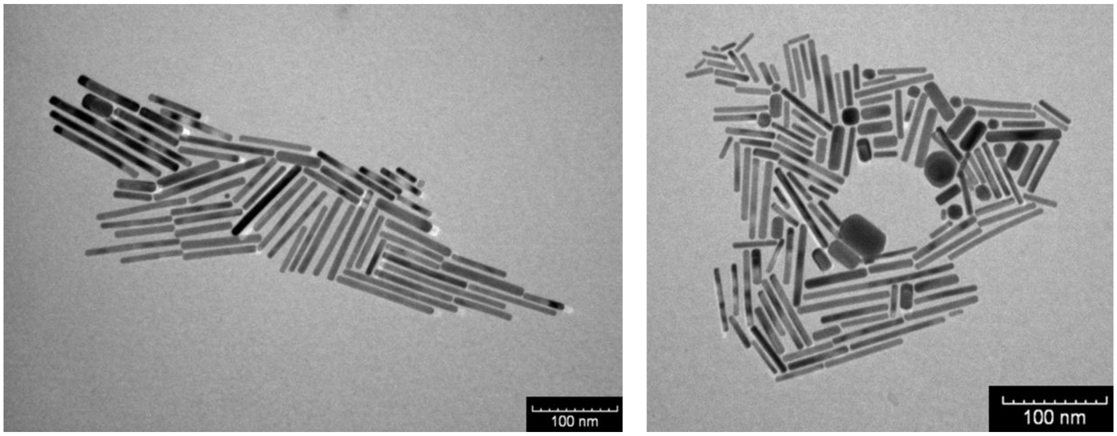

Figure S6. TEM image of Au nanorods formed at CTAB concentration of 0.05M with the addition of 100-µl toluene to the Au growth solution prior to the addition of the Au seed solution.

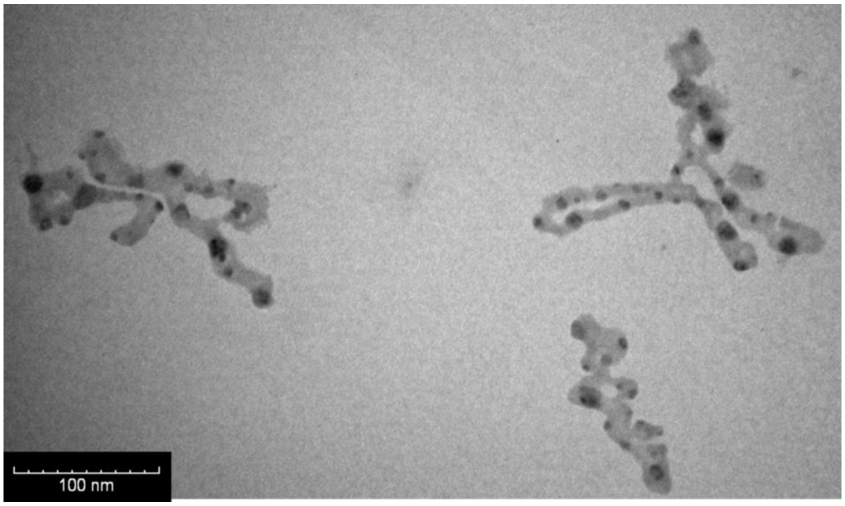

Figure S7. TEM image of Au nanostructures with irregular shapes formed at low Ag^+ ion concentration (i.e., 0.04 mM by adding 40-µl 0.01M Ag^+ solution to a 10-ml Au growth solution) with the addition of 100-µl toluene. The concentration of CTAB was 0.1M.

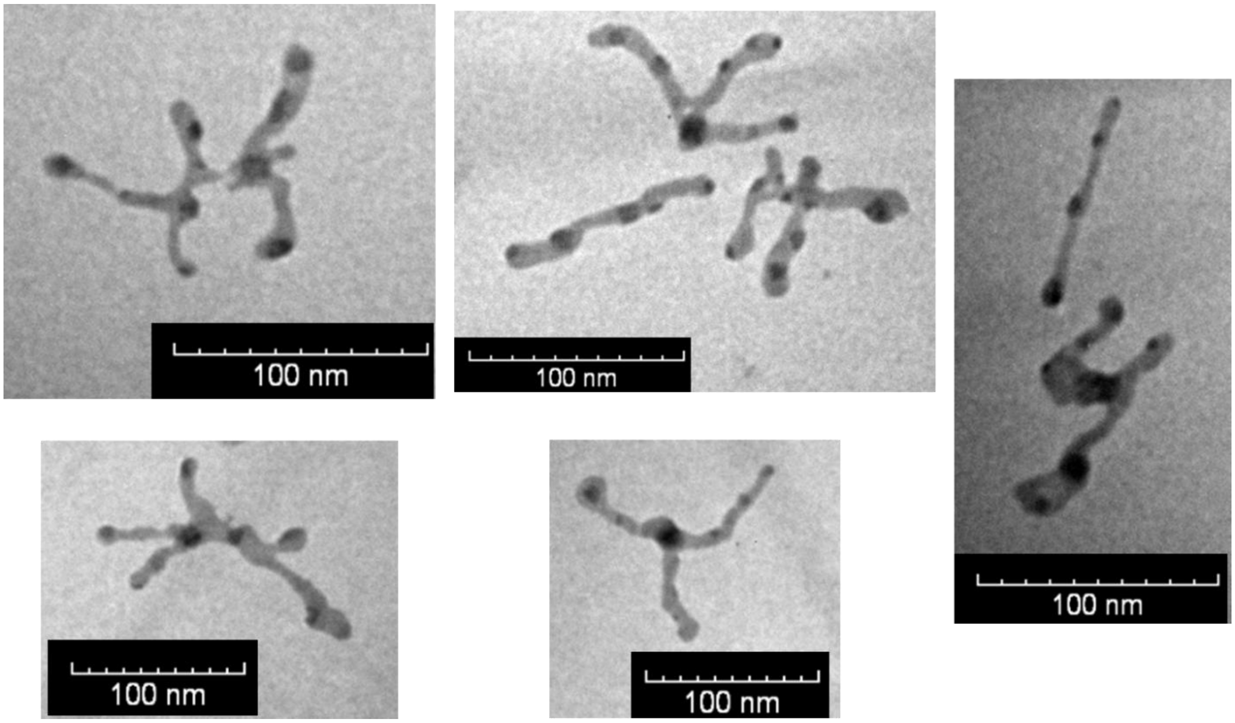

Figure S8. TEM image of Au nanostructures with irregular shapes formed at low Ag^+ ion concentration (i.e., 0.04 mM by adding 40-µl 0.01M Ag^+ solution to a 10-ml Au growth solution) with the addition of 100-µl toluene. The concentration of CTAB was 0.1M.

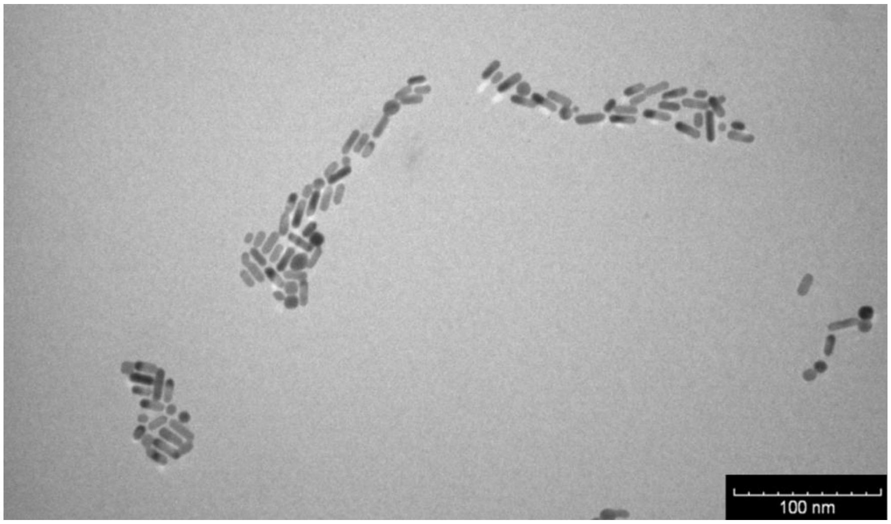

Figure S9. TEM image of short Au nanorods formed at the low Ag^+ concentration (i.e., 0.04 mM) without the addition of toluene.

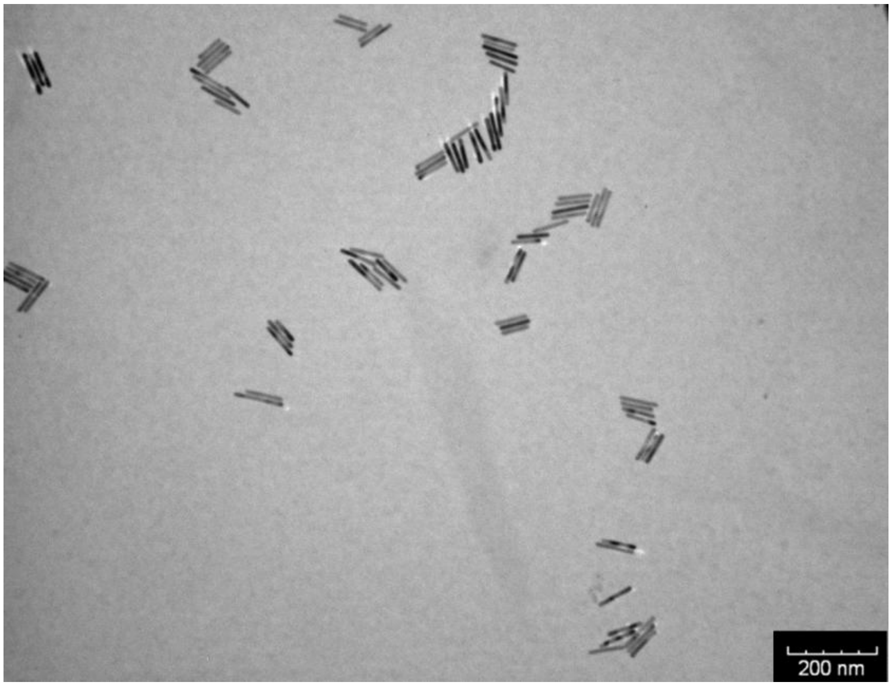

Figure S10. TEM image of Au nanorods formed on the bottom layer of solution with the addition of 100 µl toluene to the Au growth solution after adding the Au seed solution. The mixed solution was only gently shaken by hands without stirring. The growth solution was prepared by mixing 10 ml 0.1M CTAB aqueous solution with 0.5 ml 10 mM $HAuCl_4$, 40 µl 0.1 M $AgNO_3$ and 0.5 ml 0.1 M hydroquinone aqueous solution.

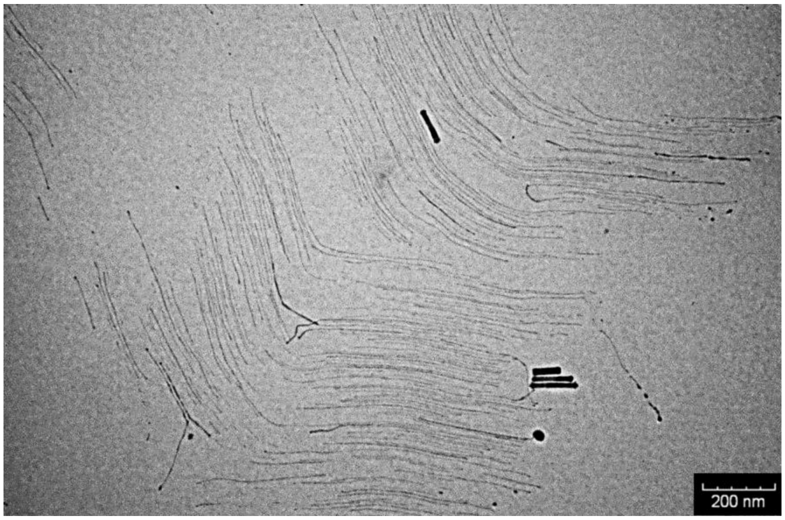

Figure S11. TEM image of Au nanorods formed on the upper layer of solution with the addition of 100-μl toluene to the Au growth solution after adding the Au seed solution. The mixed solution was only gently shaken by hands without stirring. The growth solution was prepared by mixing 10 ml 0.1M CTAB aqueous solution with 0.5 ml 10 mM $HAuCl_4$, 40 μl 0.1 M $AgNO_3$ and 0.5 ml 0.1 M hydroquinone aqueous solution.

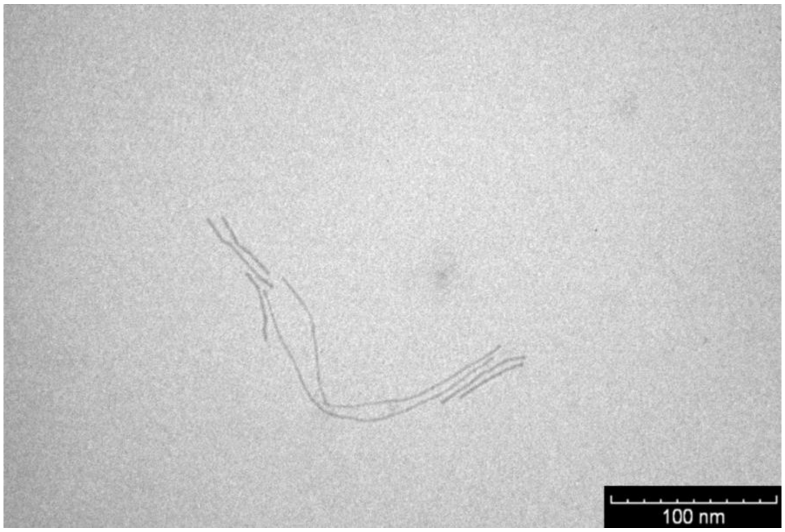

Figure S12. TEM image of ultrathin Au nanowires formed by adding a 80-μl chloroform to the Au growth solution after adding the Au seed solution. The growth solution was prepared by mixing 10 ml 0.1M CTAB aqueous solution with 0.5 ml 10 mM $HAuCl_4$, 40 μl 0.1 M $AgNO_3$ and 0.5 ml 0.1 M hydroquinone aqueous solution.
